# Potency testing of mesenchymal stromal cell growth expanded in human platelet lysate from different human tissues

**DOI:** 10.1186/s13287-016-0383-3

**Published:** 2016-08-25

**Authors:** R. Fazzina, P. Iudicone, D. Fioravanti, G. Bonanno, P. Totta, I. G. Zizzari, L. Pierelli

**Affiliations:** 1 InScientiaFides Foundation, San Marino, Republic of San Marino; 2 Immunohematology and Transfusion Medicine, San Camillo Forlanini Hospital, Rome, Italy; 3 Department of Experimental Medicine, Sapienza University, Rome, Italy; 4 Institute of Gynecology and Obstetrics, Sacred Heart Catholic University, Rome, Italy; 5 Futura Relife Srl, Rome, Italy

**Keywords:** Mesenchymal stromal cells, Culture standardization, Platelet lysate, Proliferative potential, Umbilical cord tissue, Adipose tissue, Bone marrow

## Abstract

**Background:**

Mesenchymal stromal cells (MSCs) have been largely investigated, in the past decade, as potential therapeutic strategies for various acute and chronic pathological conditions. MSCs isolated from different sources, such as bone marrow (BM), umbilical cord tissue (UCT) and adipose tissue (AT), share many biological features, although they may show some differences on cumulative yield, proliferative ability and differentiation potential. The standardization of MSCs growth and their functional amplification is a mandatory objective of cell therapies. The aim of this study was to evaluate the cumulative yield and the ex vivo amplification potential of MSCs obtained from various sources and different subjects, using defined culture conditions with a standardized platelet lysate (PL) as growth stimulus.

**Methods:**

MSCs isolated from BM, UCT and AT and expanded in human PL were compared in terms of cumulative yield and growth potential per gram of starting tissue. MSCs morphology, phenotype, differentiation potential, and immunomodulatory properties were also investigated to evaluate their biological characteristics.

**Results:**

The use of standardized PL-based culture conditions resulted in a very low variability of MSC growth. Our data showed that AT has the greater capacity to generate MSC per gram of initial tissue, compared to BM and UCT. However, UCT-MSCs replicated faster than AT-MSCs and BM-MSCs, revealing a greater proliferation capacity of this source irrespective of its lower MSC yield. All MSCs exhibited the typical MSC phenotype and the ability to differentiate into all mesodermal lineages, while BM-MSCs showed the most prominent immunosuppressive effect in vitro.

**Conclusions:**

The adoption of standardized culture conditions may help researchers and clinicians to reveal particular characteristics and inter-individual variability of MSCs sourced from different tissues. These data will be beneficial to set the standards for tissue collection and MSCs clinical-scale expansion both for cell banking and for cell-based therapy settings.

## Background

Mesenchymal stromal cells (MSCs) comprise a heterogeneous population of multipotent progenitors with multiple biological properties, including a broad differentiation potential, the ability to secrete paracrine factors, a low immunogenicity and an immunosuppressive activity. These specific characteristics make them ideal candidates for cell therapy [1–3]. MSCs were initially isolated and characterized from bone marrow (BM), but can also be found in other tissues, including adipose tissue (AT), umbilical cord tissue (UCT), dental pulp, amniotic fluid, placentae, synovial membranes, skeletal muscle, dermal tissue, and umbilical cord blood [4–6]. Based on the minimal criteria stated by the International Society of Cell Therapy (ISCT), human MSCs can be identified by the adherence to plastic, the expression of CD105, CD73 and CD90 surface antigens, the lack of hematopoietic markers (CD34, CD45, CD14 and HLA-DR), and the ability to differentiate into tissues of mesodermal origin, such as adipocytes, chondroblasts and osteoblasts [7, 8]. The prevailing view is that MSCs, irrespective of their in vivo source, exert their reparative function mostly through paracrine effects rather than by differentiation into specialized cells within the injured tissue. Indeed, it has recently come to light that MSCs secrete a wide variety of cytokines, chemokines and growth factors with immunomodulatory, angiogenic, anti-inflammatory and anti-apoptotic activity [9–14]. Moreover, MSCs exert an immunomodulatory function through the suppression of T cell proliferation, the promotion of regulatory T cell expansion and the secretion of immunosuppressive substances, protecting the injured organ from autoimmune reactions [15–18]. For these properties MSCs have been investigated in many preclinical and clinical trials in various fields, including plastic surgery, orthopedics, cardiology, neurology and hematology. In particular, MSCs have been found to be effective in the treatment of cartilage and bone defects, acute and chronic graft versus host disease (GVHD), chronic wounds, type I diabetes, rheumatoid arthritis, Crohn’s disease, multiple sclerosis, spinal cord injury, osteoarthritis, myocardial infarction and liver failure [19–31]. Although BM-MSCs were the first MSCs identified and are, therefore, the best characterized, the invasive and painful harvesting process, the low cell yield and the lower proliferation ability in standard culture conditions compared to MSCs isolated from other sources such as UCT and AT [32–34] are known limitations associated with their use. UCT and AT represent two alternative valuable sources of MSCs, easily accessible with minimally invasive procedures and reduced risks for the donor [5, 16, 33, 35–37]. AT is a convenient, abundant and readily available source of MSCs (AT-MSCs), containing 500-fold more cells per gram of tissue compared to BM. Large adipose samples can be harvested from multiple sites, allowing to obtain millions of MSCs from a single individual [38, 39]. Similarly, human UCT-MSCs (umbilical cord tissue MSCs) can be easily collected after the birth of an infant. UCT-MSCs present multipotent properties, between embryonic and adult stem cells, and exhibit a faster proliferation rate and lower immunogenicity compared to MSCs from adult tissues [5, 12, 20, 21, 34–37, 40–46]. The medium used for the MSCs culture and propagation is crucial for the safety and efficacy of MSCs and ideally should maintain their phenotype, differentiation potential and functionality during multiple passages. In the majority of studies involving MSCs, comparative analyses between different MSCs types have been done in medium supplemented with fetal bovine serum (FBS), which presents several disadvantages both for the high batch-to-batch variability, that can lead to low quality and scarce reproducibility of culture performance, and for the low safety for cell therapy use, due to the risks of xenogeneic immune reactions against bovine antigens and animal pathogen transmissions [47, 48]. To date, several human supplements have been tested as alternatives to FBS for MSC culture. In particular, several reports have demonstrated successful use of human platelet lysate (PL) as a replacement for FBS to promote growth and proliferation of MSCs without altering their phenotypic and functional characteristics [49–55]. Recently, our group has developed a protocol for the production of a standardized pathogen-free human PL for clinical-grade expansion of BM-MSCs [56]. Moreover, we have also confirmed that PL is a feasible FBS substitute for supporting growth and propagation of UCT-MSCs and human cell lines [57, 58]. The availability of defined culture conditions, optimized by a very standardized growth stimulus such as our PL, is a unique opportunity to provide a qualified and reliable growth assay for each relevant tissue with mesodermal capacity. These very standardized culture conditions may help to identify both source property and inter-individual variability and to reveal the proliferative potential per gram of collected tissue. In this study, we have compared MSCs isolated from BM, UCT and AT in terms of growth potential per gram of tissue, confirming their morphology, phenotype, differentiation and immunological capacity in standardized culture conditions. These data will be very useful to set the acceptance criteria for tissue collection and banking. To the best of our knowledge, no work has so far been reported that evaluates and compares the cumulative yield of MSCs isolated from different tissues and from different subjects by a standardized culture method.

## Methods

### Platelet lysate production and lot testing

Platelet lysates (PL, trademarked as Mesengen™ and kindly provided by Futura Relife Srl Rome, Italy) were obtained by pools of pathogen-inactivated human donor platelet concentrates as previously described [56]. PL lots were released for laboratory use only when the following criteria had been met by screened aliquots at least on BM-MSCs: (a) isolation of the first generation of MSCs within 2 weeks of culture (Passage 0: P0); (b) four fold expansion at each further passage within 1 week of culture; (c) a cumulative MSCs expansion from P0 to passage 2 (P2) equal to 35-fold or higher within 1 month of culture. Different PL lots were tested for their ability to promote the growth of MSCs derived from three tissue sources: BM, UCT and AT. For this purpose, BM-MSCs, UCT-MSCs and AT-MSCs were seeded at the same cell concentration in six-well plates and each MSC line was cultured simultaneously with eight different PL lots. The cell proliferation rate of the different MSC cultures was evaluated at two successive passages of cell expansion.

### Isolation of MSCs from bone marrow, umbilical cord and adipose tissue

All samples were obtained from healthy donors with informed consent and the study was approved by the Research Ethics Committee of San Camillo Hospital of Rome (approval date June 4, 2015). BM samples (*n* = 10) were obtained from healthy donors for allogeneic BM transplantation (six men and four women; mean age 43 ± 7 years, range 29–57). Human UCTs were collected after caesarian deliveries (*n* = 10) and human AT samples (*n* = 10) were harvested during liposuction intervention (two men and eight women; mean age 42 ± 9 years, range 28–53). The tissues were stored at 4 °C and processed within 24 hours from collection. BM-MSCs were isolated as previously described [56]. Briefly, BM-derived mononuclear cells (BM-MNC) were isolated by density gradient (Lympholyte, Cedarlaine, Burlington, ON, Canada), washed with phosphate-buffered saline (PBS, Euroclone, Pero, MI, Italy) with 0.5 % human albumin (Grifols, Sant Cugat del Valles, Barcelona, Spain) and 0.05 M EDTA (Sigma-Aldrich, St Louis, MO, USA) and counted using an automatic cell counter (ABX Pentra 400, Horiba, Irvine, CA, USA). UCT cells were isolated as previously described [58]. Briefly, whole cord tissue was washed with sterile saline solution to remove blood and blood clots from the outer layer and immersed in 0.05 % sodium hypochloride (Angelini, Rome, Italy) for 2 minutes. After washing, 10 grams of UCT were cut into small pieces with a sterile scalpel. Tissue fragments were first mechanically dissociated with gentleMACS dissociator (Miltenyi Biotech GmbH, Bergisch Gladbach, Germany) following the manufacturer's instructions and then incubated for 1.5 hours at 37 °C in a solution containing PBS with calcium and magnesium, 1 IU/ml collagenase NB4 (Serva Electrophoresis, Heidelberg, Germany) and 1 IU/ml hyaluronidase (Bioindustria, Laboratorio Italiano Medicinali, Novi Ligure, AL, Italy). After digestion, the primary umbilical cord tissue cells (UCTCs) were filtered through a 100-μm cell strainer (Miltenyi Biotech), washed with PBS with 0.5 % human albumin (Grifols) and 0.02 M ACDA and finally counted using trypan blue assay. Lipoaspirates were washed with PBS to remove contaminating blood cells and local anaesthetics. Then, the ATs were enzymatically digested with collagenase NB4 (0.15 IU per gram of tissue) (Serva Electrophoresis) at 37 °C for 40 minutes in agitation. The digested tissues were washed with PBS and 0.5 % human albumin, centrifuged at 1750 rpm for 10 minutes to separate the floating adipocytes from the pelleted primary stromal cells, identified as stromal vascular fraction (SVF). SVF cells were suspended in PBS, passed through a 100-μm mesh filter to remove clots and counted using an automatic cell counter (ABX Pentra 400, Horiba).

### MSCs selection and expansion

Primary BM-MNCs, UCTCs and SVF cells were plated in culture medium (CM) consisting of α-Minimum Essential Medium (α-MEM) (Euroclone) supplemented with 10 % PL, 2 IU/ml heparin, 2 mM L-glutamine 100 U/ml penicillin and 100 μg/ml streptomycin (Euroclone). CM was replaced every 3 days. After 48 h the medium was changed and the non-adherent cells were collected, centrifuged and replated in a new flask. CM for all cultures was replaced every 3 days. Once the cultures reached 80 % confluence (P0), cells were harvested by Trypsin-EDTA treatment (Euroclone), counted with a Neubauer chamber and subcultured at 2 × 10^3^ cells per cm^2^ until Passage 2 (P2). Proliferation rate was determined by calculating total cell yield and population doubling time (PDT). The total number of cells at each passage was calculated as a ratio of number of cells harvested to number of cell seeded, multiplied by the total number of cells from the previous passage. MSCs total yield was calculated per gram of processed tissue at both Passage 1 (P1) and P2. PDTs were calculated from cell counts with the following mathematical equation: PDT: T (log2)/log (Y) - log(X) where (X) indicates cells at seeding, (Y) the harvested cells and (T) the culture time (days).

### MSC phenotype analysis by flow cytometry

For phenotypic analysis BM-MSCs, UCT-MSCs and AT-MSCs were harvested and stained for 15 minutes at room temperature with the following monoclonal antibodies (mAbs): anti-human CD90 APC, CD73 PE, CD34 PE, CD200 PE, CD273 APC, CD274 PE, CD71 FITC, CD44 PE (BD Pharmingen, Heidelberg, Germany), anti-human HLA-DR PE, HLA-A,B,C FITC, CD144 (VE-cadherin) APC, CD31 FITC, CD105 PE (Biolegend, San Diego, CA, USA), CD45 FITC (Tonbo, Biosciences, San Diego, CA, USA) or with the appropriate fluorochrome-conjugated isotype-matched mAbs to establish background fluorescence. After incubation cells were washed with PBS, centrifuged at 1400 RPM for 5 minutes and suspended in PBS for flow-cytometry analysis. Samples were acquired using a FACSCalibur (Becton Dickinson, San Josè, CA, USA) and the data were analysed with CellQuest software (Becton Dickinson).

### Osteogenic, chondrogenic and adipogenic differentiation assays

The MSCs osteogenic differentiation was induced using the Human Mesenchymal Stem Cell Osteogenic Differentiation Medium BulletKit (Lonza, Basel, Switzerland) according to the manufacturer's instructions. Briefly, 3 × 10^4^ cells were seeded into a 35-mm cell culture dish in CM and incubated at 37 °C in a humidified atmosphere with 5 % CO_2_. After 24 hours the medium was replaced with the Osteogenic Induction Medium and changed every 3–4 days. After 3 weeks of differentiation, cells were fixed with methanol and stained with alizarin red S (Sigma-Aldrich) according to the manufacturer’s instructions. The MSCs adipogenic differentiation was induced using the Human Mesenchymal Stem Cell Adipogenic Differentiation Medium BulletKit™ (Lonza) according to the manufacturer's instructions. Briefly, 2 × 10^5^ cells were seeded into a 35-mm cell culture dish in CM and incubated at 37 °C in a humidified atmosphere with 5 % CO_2_ until confluence. At 100 % confluence the medium was replaced with the Adipogenic Induction Medium and after 3 days the medium was replaced with the Adipogenic Maintenance Medium. Three cycles of induction/maintenance were performed. After 3 weeks cells were washed twice with deionized H_2_O, fixed with 4 % of formaldehyde solution for 10 minutes and stained with Oil Red O (Sigma-Aldrich) to detect adipocytes. The MSCs differentiation into chondrogenic lineage was performed using Human Mesenchymal Stem Cell Chondrogenic Differentiation Medium BulletKit™ (Lonza) according to the manufacturer's instructions. Briefly, a micromass culture of UCT-MSCs was prepared resuspending 2.5 × 10^5^ cells in Chondrogenic Differentiation Medium (Lonza) into a polypropylene conical tube. Tubes were incubated at 37 °C in a humidified atmosphere with 5 % CO_2_ and the differentiation medium was replaced every 3 days. After 24 days the differentiated chondrocytes, which formed three-dimensional clusters, termed chondrocyte nodules, were fixed in 4 % formalin and incubated overnight at room temperature. Nodules were dehydrated with ethanol dilution series followed by 30 minutes incubation in xylol and then embedded in paraffin at 58 °C. Three-micron-thick tissue sections were obtained with a microtome. Chondrocyte differentiation was detected by observing eosin/haematoxylin (Sigma-Aldrich) staining of extracellular matrix.

### MSC immunomodulation assay (lymphocyte proliferation assay)

The immunoregulatory effect of BM-MSCs, UCT-MSCs and AT-MSCs was evaluated in two experiments by co-culturing MSCs and peripheral blood mononuclear cells (PBMCs) using a carboxy fluorescein diacetate succinimidyl ester (CFSE)-based proliferation assay (CSFE: Invitrogen, Waltham, MA, USA). BM-MSCs, UCT-MSCs and AT-MSCs were irradiated with 50 Gy and plated in 0.1 % gelatin (Merck Millipore, Billerica, MA, USA) pre-coated 24-well plates at different cell concentrations: 2.5 × 10^5^ cells per well (MSCs/PBMC ratio 1:4) and 1.25 × 10^5^ cells per well (MSCs/PBMC ratio 1:8). MSCs were plated in duplicate in 2 ml of CM and allowed to adhere to the plate for 24 h in the presence of 50 ng/μl interferon-γ (INF-γ; Miltenyi Biotech). Human PBMCs were obtained from healthy blood donors after written informed consent. PBMCs were isolated by density gradient centrifugation (Lympholyte®-H, Cedarlane, Hornby, ON, Canada), washed in PBS supplemented with FBS 5 % (Euroclone) and counted by an automatic cell counter (ABX Pentra 400, Horiba). PBMCs were fluorescent-labelled with 2 μM CFSE by incubating for 15 minutes at 37 °C in the dark. Quench staining was performed on ice for 5 minutes by adding 5 volumes of ice-cold PBS supplemented with 20 % FBS. Cells were then washed three times with cold PBS plus 10 % FBS and analysed for CFSE staining. 1 × 10^6^ CFSE-stained PBMCs were added to the wells previously seeded with 2.5 × 10^5^ or 1.25 × 10^5^ adherent MSCs to obtain 1:4 and 1:8 MSCs/PBMCs ratios respectively. Co-cultures were incubated in CM supplemented with 50 ng/ml pure anti-CD3 functional grade monoclonal antibody (mAb) OKT3 (Miltenyi Biotech) to stimulate T lymphocytes, and with 300 IU/mL interleukin-2 (IL-2, Miltenyi Biotech) to sustain T cell proliferation. The co-culture was carried out using 24-well plates (contact culture) or transwell culture chambers (Corning Inc/Costar, Corning, NY, USA), in which the PBMCs and MSCs were physically separated by a membrane permeable for soluble factors. The PBMCs stained with CSFE and cultured with anti-CD3 and IL-2 in the absence of MSCs were used as control for lymphocyte proliferation. Cultures were incubated at 37 °C in 5 % CO_2_ and analysed after 72, 96 and 120 hours. In a CFSE-based assay, following proliferative stimulus, the stained cells undergo mitotic events which generate a CFSE dilution profile. Generational boundaries identify adjacent daughter peaks, which are used to calculate the percentage of proliferating cells falling in each daughter peak. At the established times of cell culture, aliquots of the CFSE-labelled cells were harvested, washed with PBS supplemented with 1 % FBS and incubated for 10 minutes in the dark with 7-amino-actinomycin D (7-AAD, BD Biosciences, San Jose, CA, USA) to exclude dead cells. The flow cytometry analysis allowed estimation of the number of proliferating cells with respect to the starting cell number. The levels of lymphocyte proliferation observed in the co-cultures of CFSE-PBMCs plus MSCs were normalized to the control without MSC, which was set as 100 % proliferation. The inhibition of cell proliferation was expressed as the percentage of total proliferating cells.

### Statistical analysis

All comparisons were performed by one-way ANOVA and Scheffe test as post hoc comparison. A *p* < 0.05 was considered significant. All tests were performed by StatPlus:mac Pro, version 6.0.3 (AnalystSoft Inc., Walnut, CA, USA).

## Results

### PL preparations: assessment of reproducibility

**Fig. 1:**
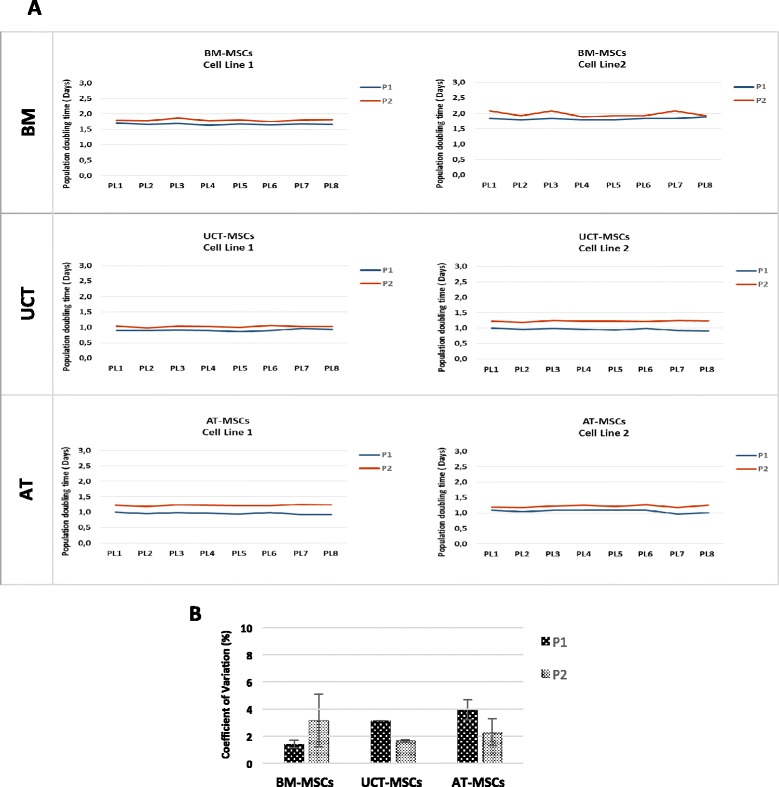
Platelet lysate lot testing. **a** MSCs growth assay with different platelet lysate lots. Two MSCs cell lines for each source (BM, UCT, AT) were used to test the growth promotion ability and variability of eight different PL lots (PL1–PL8). MSCs proliferation was evaluated at passage 1 (P1) and passage 2 (P2). **b** Coefficient of variation percentage related to the eight different PL lots (PL1–PL8) in BM-MSCs, UCT-MSCs, and AT-MSCs cultures at P1 and P2. Results are expressed as mean ± SD (standard deviation). (F = 0.479 and *p* = 0.847 at ANOVA and *p* > 0.900 for any post hoc comparison at Scheffe test) *AT* adipose tissue, *BM* bone marrow, *MSCs* mesenchymal stromal cells, *UCT* umbilical cord tissue

Different PL lots, obtained according to standard protocols previously reported and released in the presence of criteria indicated in “Material and Methods”, were evaluated once again for the ability to sustain the growth of MSCs from different sources. Figure 1a shows that eight lots of PL (PL1–PL8) supported an identical doubling capacity of individual MSC populations derived respectively from BM, UCT and AT, as revealed by the PDT measured at P1 and P2, with an average variation coefficient ever less than 4 % (Fig. 1b) (F = 0.479 and *p* = 0.847 at ANOVA and *p* > 0.900 for any post hoc comparison at Scheffe test). These data confirm that the different PL lots were manufactured to a consistent reproducible quality ensuring reliable standardized culture conditions when evaluating the growth potential of MSC from different sources.

### Potential of different tissues in generating MSCs

**Fig. 2:**
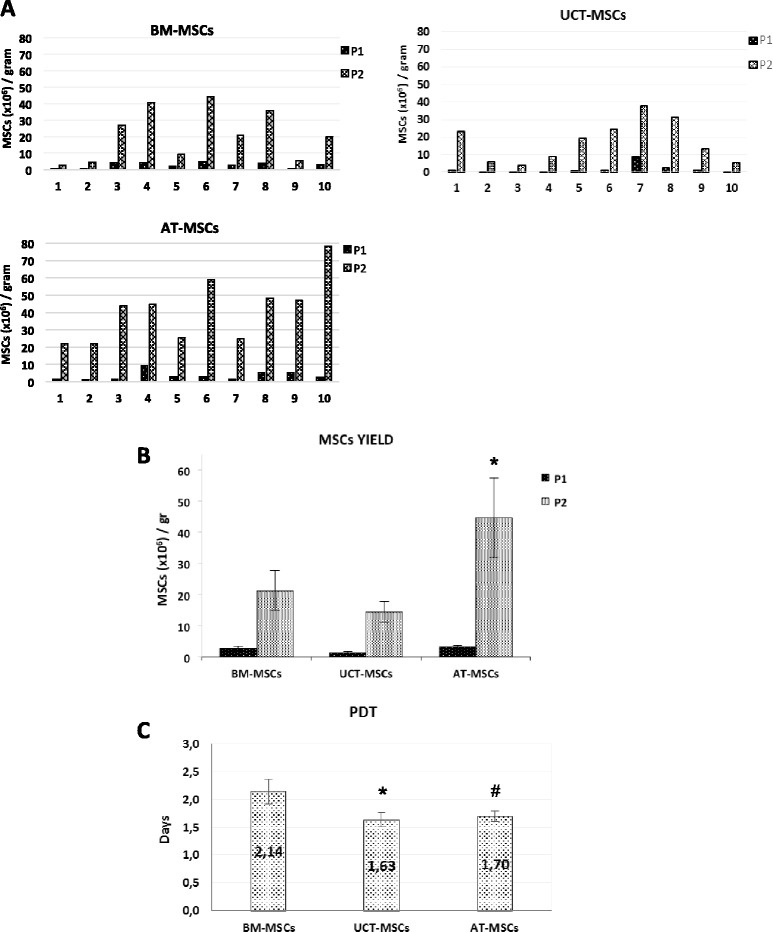
Expansion potential of MSCs from BM, UCT and AT. **a** The expansion of MSCs from ten samples of each source (BM, UCT, AT) revealed a high inter-individual variability in the amount of MSCs obtained per gram of initial tissue, which ranged between 2 × 10^6^ and 80 × 10^6^ at Passage 2 (P2). **b** Comparison of the average of MSCs cumulative cell yield per gram of initial tissue (BM, UCT, AT) at Passage 1 (P1) and P2. Results were represented as mean ± SEM (standard error of the mean) (F = 19.712 and *p* < 0.000 at ANOVA; *p = 0.008 for AT vs BM and *p* = 0.001 for AT vs UCT at Scheffe test). **c** Comparison of the population doubling time (PTD) mean calculated at P1 and P2 of MSCs populations derived from the different tissues. Data are shown as means ± SD of ten samples processed for each tissue (F = 3.960 and *p* = 0.002 at ANOVA and *p = 0.042 for UCT vs BM and ^#^ *p* = 0.050 for AT vs BM at Scheffe test for P2). *AT* adipose tissue, *BM* bone marrow, *MSCs* mesenchymal stromal cells, *UCT* umbilical cord tissue

The consistent and reproducible results observed in terms of growth (as PDT), using several PL lots on the same MSC population from all sources, allowed us to further investigate the yield of MSCs obtained from a given amount of BM, AT and UCT starting tissue under standardized conditions. The results of ten MSC expansions from different tissue origin revealed that each source shows a high inter-individual variability in the amount of MSCs obtained per gram of initial tissue, which ranged between 2 × 10^6^ and 80 × 10^6^ at P2 (Fig. 2a). On average, AT exhibited the greater capacity of generating MSCs (44.6 × 10^6^ ± 12.7 × 10^6^ per gram) compared to both BM (21.3 × 10^6^ ± 6.3 × 10^6^ per gram) and UCT (14.4 × 10^6^ ± 3.3 × 10^6^ per gram) (Fig. 2b) (F = 19.712 and *p* = 0.000 at ANOVA; *p* = 0.008 for AT vs BM and *p* = 0.001 for AT vs UCT at Scheffe test). Regarding the proliferation capacity of MSC populations derived from the different tissues, Fig. 2c shows the average value of PDT calculated at P1 and P2, which resulted in 2.14 ± 0.22, 1.63 ± 0.13 and 1.7 ± 0.09 days for BM-MSCs, UCT-MSCs and AT-MSC respectively (F = 3.960 and *p* = 0.002 at ANOVA and *p* = 0.042 for UCT vs BM and *p* = 0.050 for AT vs. BM at Scheffe test for P2). Our results showed that once MSC are selected from the initial primary cell population (P0), UCT-MSCs and AT-MSCs cultures replicated faster than BM-MSCs, irrespective of the MSCs yield per gram of initial tissue if standardized culture conditions are used.

### General characterization of MSCs and differentiation assay

**Fig. 3:**
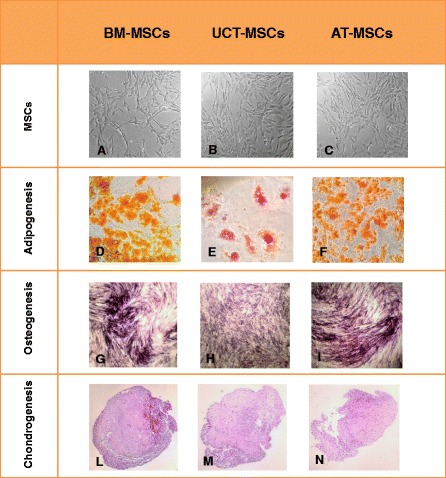
Morphology and differentiation assays of MSCs. Spindle-shaped adherent cells with MSCs morphology were observed in BM-MSCs (**a**), UCT-MSCs (**b**) and AT-MSCs (**c**) cultures. Representative images of BM-MSCs, UCT-MSCs and AT-MSCs induced to differentiate into adipogenic (**d**-**f**), osteogenic (**g**-**i**), and chondrogenic lineages (**l**-**n**). (Magnification × 100). *AT* adipose tissue, *BM* bone marrow, *MSCs* mesenchymal stromal cells, *UCT* umbilical cord tissue

MSC population isolated from BM, AT and UCT were analysed in order to evaluate cell morphology and differentiation potential. Spindle-shaped adherent cells with MSC morphology were observed among the three populations (Fig. 3a–c). When we analysed and compared the ability of UCT-MSCs, AT-MSCs and BM-MSCs to differentiate into the three mesenchymal lineages, we found that all MSC populations had the ability to differentiate towards adipocytes, osteoblasts and chondrocytes (Fig. 3). In the adipogenic induction, many large, flattened, often oval cells appeared in the culture, showing large lipid droplet accumulation positive for Oil Red O staining, while the non-stimulated cells maintained their original spindle-shaped morphology and the formation of lipid granules was not observed (Fig. 3d–f). After 3 weeks under osteogenic stimuli all MSCs showed typical osteogenic morphological features and calcium deposits revealed by Alizarin Red S staining, while the unstimulated MSCs did not present Ca^++^ deposits (Fig. 3g–i). Under chondrogenic stimuli MSCs showed cell condensation in nodule-like structures and a high intensity of glycosaminoglican production from cell aggregates, revealed by eosin-haematoxylin staining (Fig. 3l–n). No micromass formation was observed in non-conditioned MSCs. The quantitative analysis of the different cultures revealed that UCT-MSCs showed a weaker differentiation potential compared to AT-MSCs and BM-MSCs. Indeed, in all differentiation assays, UCT-MSCs showed both a less percentage of differentiated cells (about 30 % less, on average) and a weaker staining intensity.

### MSC surface antigen profile

**Fig. 4:**
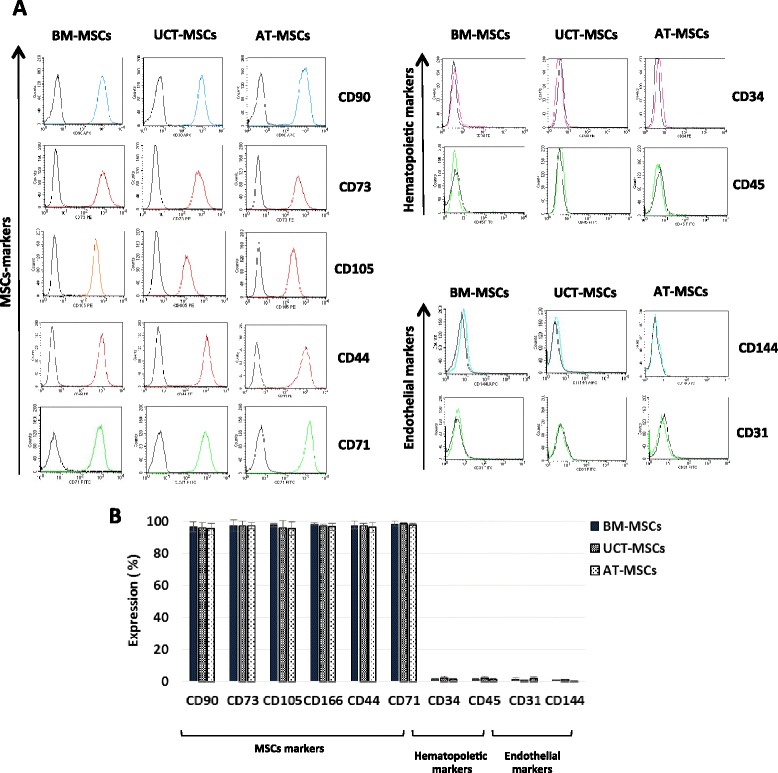
Flow cytometric analysis of MSCs phenotype. **a** Histograms showing the MSCs, hematopoietic and endothelial surface antigen expression of BM-MSCs, UCT-MSCs and AT-MSCs. One representative MSC sample for each source is shown. **b** Quantitative expression of MSCs, hematopoietic and endothelial antigens measured by flow cytometry. Results are expressed as mean ± SD (standard deviation) of ten samples processed for each tissue. *AT* adipose tissue, *BM* bone marrow, *MSCs* mesenchymal stromal cells, *UCT* umbilical cord tissue

**Fig. 5:**
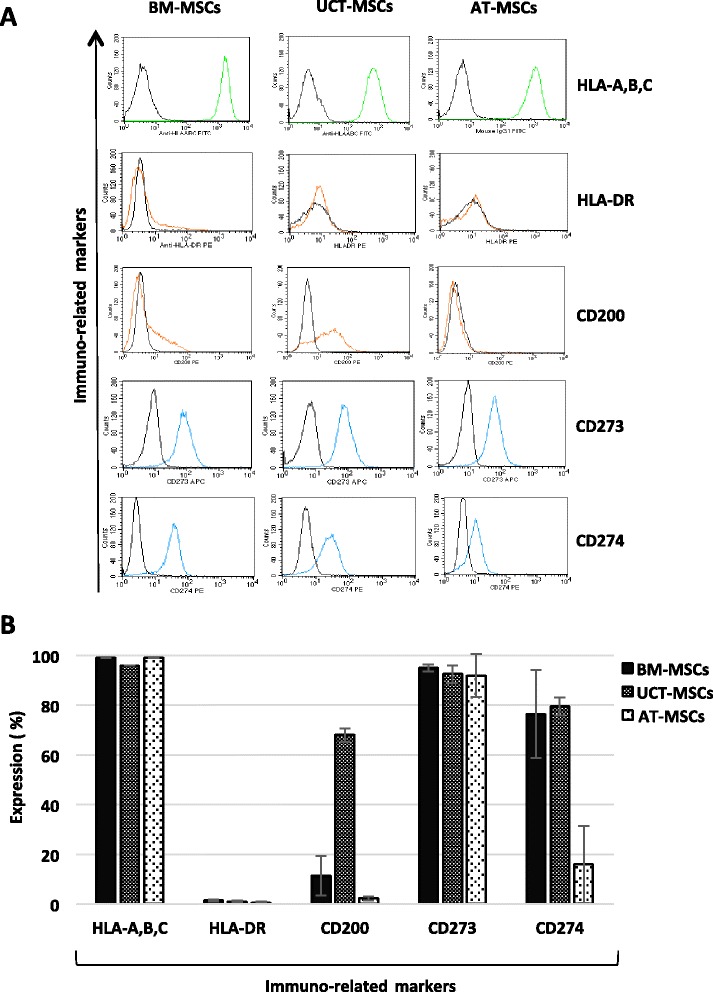
Immune-related markers. **a** Histograms showing the immune-related antigen expression of BM-MSCs, UCT-MSCs and AT-MSCs. One representative MSC sample for each source is shown. **b** Quantitative expression of immune-related markers measured by flow cytometry. Results are expressed as mean ± SD (standard deviation) of five samples processed for each tissue. *AT* adipose tissue, *BM* bone marrow, *MSCs* mesenchymal stromal cells, *UCT* umbilical cord tissue

MSCs derived from all tissues exhibited a typical MSC phenotype, as defined by ISCT, being strongly positive for CD73, CD105, CD90, CD44 and CD71 and negative for the hematopoietic markers CD34, CD45 and for the typical endothelial antigens CD31 and CD144 (Fig. 4a, b). The flow-cytometry analysis of immune-related markers confirmed the constitutive expression of HLA-A,B,C antigens and the absence of HLA-DR. Low levels of CD200 were found in most UCT-MSCs and in a small subset of BM-MSCs, while it was absent in the majority of AT-MSCs. The surface protein CD273 was highly expressed on MSCs from all tissues, while CD274 was revealed mostly on the surface of BM-MSCs and UCT-MSCs, whereas it exhibited low expression in AT-MSCs (Fig. 5a, b). Our results are in accordance to published data, revealing that immune-related markers CD200, CD273 and CD274 are differently expressed on MSCs depending on the tissue of origin and that, among the different MSC sources, UCT-MSCs express these markers in the greatest proportion. These differences may affect the immunological properties of each MSCs population and may explain the lower immunogenicity of UCT-MSCs compared to MSCs derived from other sources [18]. On the other hand, it is worth noting that BM-MSCs may have a putative advantage in terms of low immunogenicity as compared to AT-MSCs, due to relevant expression of at least the CD274 antigen.

### MSCs immunomodulation assay

**Fig. 6:**
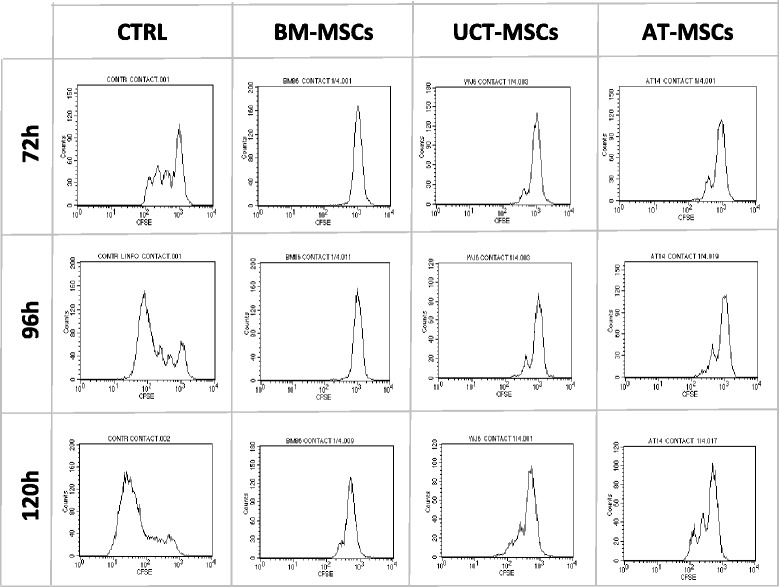
CFSE dilution profile in CFSE-based lymphocyte proliferation assay. Histograms representing the distribution of proliferating lymphocytes with respect to CFSE expression level. When labelled cells undergo cell division, the CFSE fluorescence intensity is reduced by 50 %, generating new peaks on the left side of the initial peak of dye intensity. CFSE dilution profiles of total proliferating lymphocytes at 72 h, 96 h and 120 h in the absence (CTRL) or in the presence of BM-MSCs, UCT-MSCs and AT-MSCs are shown. *AT* adipose tissue, *BM* bone marrow, *CTRL* control, *MSCs* mesenchymal stromal cells, *UCT* umbilical cord tissue

**Fig. 7:**
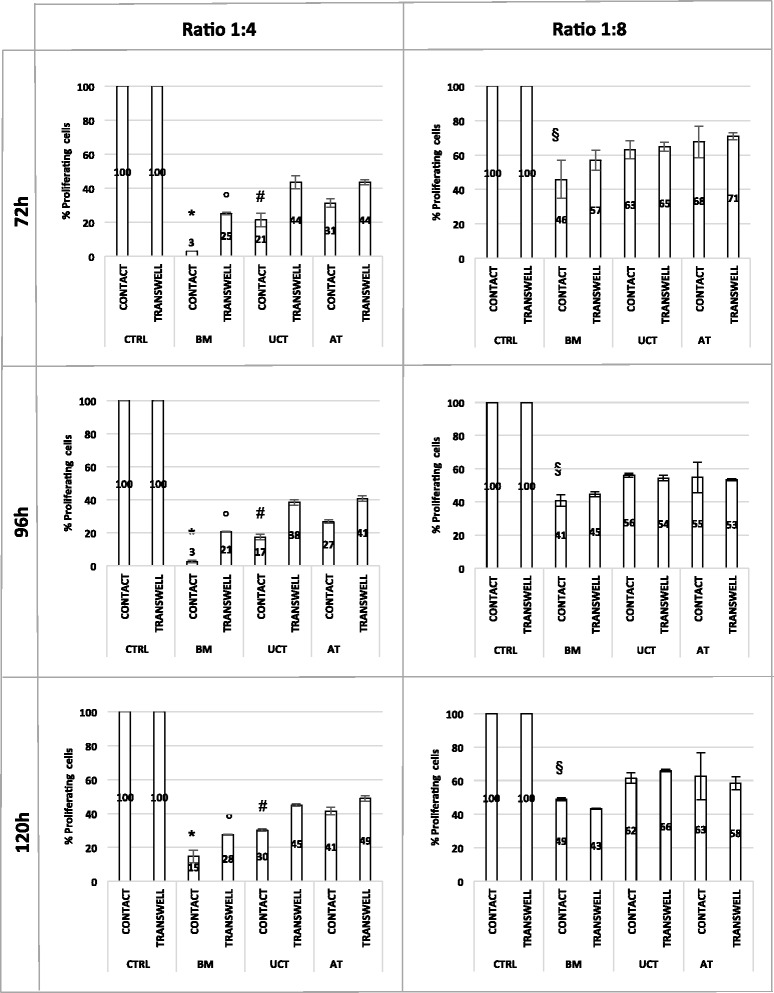
MSC immunosuppressive effect on lymphocyte proliferation. Data show the percentages of residual proliferative capacity of stimulated lymphocytes in the absence (CTRL) or in the presence of BM-MSCs, UCT-MSCs and AT-MSCs at ratio 1:4 and 1:8 (MSCs:PBMCs), either in cell-cell contact or in transwell system, evaluated at 72 h, 96 h and 120 h. (F = 29.025 and *p* = 0.000 at ANOVA and ^*^ *p* = 0.000 for BM vs AT, ^*^ *p* = 0.001 for BM vs UCT, ^#^ *p* = 0.029 for AT vs UCT at Scheffe test for 1:4 ratio in contact cultures; F = 53.301 and *p* = 0.000 at ANOVA and °*p* = 0.000 for BM vs AT, °*p* = 0.001 for BM vs UCT, *p* = 0.237 for AT vs UCT at Scheffe test for 1:4 ratio for transwell cultures; F = 6.57 and *p* = 0.008 at ANOVA and *p* = 0.152 for BM vs AT, ^§^ *p* = 0.009 for BM vs UCT, *p* = 0.328 for AT vs UCT at Scheffe test for 1:8 ratio in contact cultures; F = 2.86 and *p* = 0.088 at ANOVA and *p* = 0.151 for BM vs AT, *p* = 0.152 for BM vs UCT, *p* = 0.981 for AT vs UCT at Scheffe test for 1:8 ratio in transwell cultures). *AT* adipose tissue, *BM* bone marrow, *CTRL* control, *UCT* umbilical cord tissue

To test whether MSCs obtained from various sources could exert an immunoregulatory activity, CFSE-labelled PBMCs, exposed to lymphocyte proliferation stimulus, were co-cultured with irradiated MSCs. In the CFSE proliferation assay, the flow-cytometry analysis generates a histogram representing the distribution of cells with respect to the CFSE expression level. Following each cell division, the equal distribution of the CFSE to progeny cells results in a halving of the fluorescence of daughter cells, which specify the percentages of live cells that have undergone cell divisions. To estimate the levels of lymphocyte proliferation in the co-cultures with MSCs, it was assumed that lymphocyte growth in the absence of MSCs were 100 % proliferating (control). The residual proliferative capacity of stimulated lymphocytes in the presence of MSCs was then normalized to this control. Figure 6 shows typical CFSE dilution profiles in the absence of MSCs (control) or in the presence of MSCs obtained from all sources. Our data show that in the presence of MSCs most proliferating lymphocytes fall into the first-generation peak and do not move to the next ones, as a result of MSC immunosuppressive action. In particular, we found that BM-MSCs strongly inhibited lymphocyte proliferation within the entire culture time (120 h), while the immunosuppressive effect was less marked, but still remarkable, in UCT-MSCs and AT-MSCs co-cultures (Fig. 7). Moreover, it is worth mentioning that the ratio 1:4 was more effective than 1:8 and that cell-to-cell contact was required to induce the highest immunosuppressive effect. Indeed, at the ratio of 1:4, in the cultures established in a cell-to-cell contact system, the percentage of residual proliferating lymphocytes in the presence of BM-MSCs resulted in 3 % at 72 h and 96 h, and 15 % at 120 h, whereas it was 21 %, 17 % and 30 % in the presence of UCT-MSCs and 31 %, 27 % and 41 % with AT-MSCs respectively (Fig. 7) (F = 29.025 and *p* = 0.000 at ANOVA and *p* = 0.000 for BM vs AT, *p* = 0.001 for BM vs UCT, *p* = 0.029 for AT vs UCT at Scheffe test). AT-MSCS and UCT-MSCs produced a lower proliferative reduction of responding lymphocytes, especially when tested in transwell cultures (F = 53.301 and *p* = 0.000 at ANOVA and *p* = 0.000 for BM vs AT, *p* = 0.001 for BM vs UCT, *p* = 0.237 for AT vs UCT at Scheffe test). Overall, the 1:8 ratio produced lower reduction of proliferation in all sources and samples tested (F = 6.57 and *p* = 0.008 at ANOVA and *p* = 0.152 for BM vs AT, *p* = 0.009 for BM vs UCT, *p* = 0.328 for AT vs UCT at Scheffe test for contact cultures; F = 2.86 and *p* = 0.088 at ANOVA and *p* = 0.151 for BM vs AT, *p* = 0.152 for BM vs UCT, *p* = 0.981 for AT vs UCT at Scheffe test for transwell cultures).

## Discussion

MSCs define a population of progenitor cells with low immunogenicity, ease of accessibility, broad differentiation potential and immunomodulatory effects. Due to their potential to repair and regenerate damaged tissues and organs, MSCs are being largely investigated as promising candidates for new cell-based therapies [1–3, 19, 22, 23].

In view of their rapidly increasing applications, it is essential to establish standard cell culture conditions to scale up the MSCs propagation without altering their peculiar features and functions. Moreover, in order to define standard criteria for tissue collection and banking, since cell-based therapies need significant viable numbers of cells, that in some cases are administered through an intravenous route at doses ranging from 10^6^ to 10^7^ per kg of body weight, it is important to explore both the MSCs cumulative yield potential per gram of tissue processed among different possible sources and the ex vivo amplification potential endowed with specific cell functions. MSCs can be isolated from several human tissues and, although they share many biological features, such as morphology and expression of surface antigens, there are some differences regarding proliferative rates and differentiation potential between MSCs isolated from different sources, which may be confirmed only by applying a “standardized” culture method during MSCs ex vivo expansion. In this study we attempted to provide a standardized assay to detect the expansion capacity of MSCs obtained from various sources and different subjects. We evaluated the number of MSCs obtained per gram of tissue after expansion at both Passage 1 (P1) and Passage 2 (P2), considering P2 as our growth endpoint. Each culture experiment was performed using PL, a standardized culture supplement, which has been demonstrated to have growth-promoting properties for MSCs, while maintaining their immunophenotype, differentiation potential and immunomodulatory properties. In addition, PL can be produced under Good Manufacturing Practice (GMP) conditions for clinical-scale expansion of MSCs for therapeutic applications [56, 58, 59]. In our experiments, we efficiently isolated MSCs either from BM, AT or UCT. To confirm once again the reliability of our culture conditions to generate very reproducible results in terms of MSCs growth, we evaluated the proliferative potential of the same MSC population exposed to different batches of PL preparations. Our results showed that different batches of PL supported an identical proliferation capacity of a single MSC population from both BM, UCT and AT, with an average variation coefficient ever less than 4 %. By using this standardized culture protocol, we evaluated the ability of BM, AT and UCT from different subjects to generate MSCs from a given amount of starting tissue. These cultures revealed that each source suffers from high inter-individual variability in producing MSC per gram of tissue, ranging from 2 × 10^6^ to 80 × 10^6^ at P2. The results showed that AT has the greater capacity to generate MSC per gram of tissue, compared to BM and UCT. However, taking into account the PDT mean values, our findings indicated that, once established, UCT-MSCs replicated faster than AT-MSCs and BM-MSCs, revealing a greater proliferation capacity irrespective of its lower MSC yield per gram of initial tissue. Thus, regarding the proliferation capacity of MSCs from the three tissues, our data confirmed that UCT-MSCs and AT-MSCs exhibited a higher growth rate compared to BM-MSCs in parallel cultures if they were grown in standardized conditions and not by methods that were optimized on a specific MSC source. These data appear quite reliable considering the low variability of the culture conditions employed in the experiments and are in accordance with previously published data revealing that BM-MSCs exhibit a lower proliferation rate compared to MSCs from other sources [10, 16, 33]. Regarding the general characteristics of MSCs, cells isolated from the three sources exhibited typical MSC morphology and share the expression of the classical MSC markers and the absence of hematopoietic and endothelial markers. With regard to differentiation capacity, minimal criteria defined by ISCT state that MSCs should be able to differentiate into bone, cartilage and adipose tissue under certain stimuli in vitro [7]. Our results demonstrated that all MSC populations had the ability to differentiate towards adipocytes, osteoblasts and chondrocytes. However, the qualitative and quantitative analysis of the different cultures revealed that UCT-MSCs had a weaker differentiation potential towards all lineages compared to AT-MSCs and BM-MSCs. It is widely accepted that MSCs have immunosuppressive and immunomodulatory functions [9, 12, 13, 15–17]. Due to their ability to regulate immune responses, MSCs are potential candidates for treating a wide range of immune-mediated diseases. Thus, great benefit may result from comparing the immune properties of MSCs derived from different sources to identify the best source for clinical treatment of immune-related pathological conditions.

The analysis of immune-related markers confirmed the constitutive expression of HLA-A,B,C antigens and the absence of HLA-DR in all MSCs derived from the three tissues. Furthermore, we analysed the expression of CD200, CD273 and CD274 surface antigens, which have recently been suggested to be involved in the immunoregulatory mechanism of MSCs, contributing to fetomaternal and allograft tolerance [18, 60]. We found a mild to moderate expression of CD273 and CD274 in all MSCs while CD200 was mostly expressed at low level in UCT-MSCs and in a small subset of BM-MSCs, however, was totally absent in the AT-MSCs. To further investigate the immunosuppressive properties of MSCs we examined their effect on stimulated T cell proliferation using co-culture experiments with both the contacted mix culture and the transwell system, in which the T cells and the MSCs were physically separated by a membrane permeable for soluble factors. The results demonstrated that BM-MSCs had the most prominent suppressive effect at a ratio of 1:4 during cultures established in a cell-to-cell contact system. Of note, this significant effect of BM-MSCs persisted also in non-contact cultures (transwell), still at the higher effector-target ratio. Hence, BM-MSCs seem to be the most suitable cell population to establish cell-based treatments in allo-auto aggressive disorders. However, AT-MSCS and UCT-MSCs produced a lower but still remarkable proliferative reduction of responding lymphocytes, when tested in cell-contact cultures.

## Conclusions

In this study we showed that MSCs isolated from various tissues exhibit differences in their cumulative yield, ex vivo amplification potential and immunomodulation activity. These data will be very useful to define standard criteria for tissue collection and MSCs clinical-scale expansion both for cell banking and for cell-based therapy settings. To the best of our knowledge, no work has so far been reported establishing standardized MSCs culture conditions to evaluate and compare their “potency” and their related functions from different tissues in parallel experiments.

## Abbreviations

AT: Adipose tissue
BM: Bone marrow
CFSE: Carboxy fluorescein diacetate succinimidyl ester
CM: Culture medium
FBS: Fetal bovine serum
GVHD: Graft-versus-host disease
IL-2: Interleukin-2
INF-γ: Interferon-γ
ISCT: International Society of Cell Therapy
MSCs: Mesenchymal stromal cells
P0: Passage 0
P1: Passage 1
P2: Passage 2
PBMCs: Peripheral blood mononuclear cells
PBS: Phosphate-buffered saline
PDT: Population doubling time
PL: Platelet lysate
UCT: Umbilical cord tissue
α-MEM: α-Minimum Essential Medium
